# Systemic epidermal nevus with involvement of the oral mucosa due to *FGFR3 *mutation

**DOI:** 10.1186/1471-2350-12-79

**Published:** 2011-06-05

**Authors:** Anette Bygum, Christina R Fagerberg, Ole J Clemmensen, Britta Fiebig, Christian Hafner

**Affiliations:** 1 Department of Dermatology and Allergy Centre, Odense University Hospital, 5000 Odense, Denmark; 2 Department of Clinical Genetics, Odense University Hospital, 5000 Odense, Denmark; 3 Department of Clinical Pathology, Odense University Hospital, 5000 Odense, Denmark; 4 Institute of Human Genetics, University of Regensburg, 93042 Regensburg, Germany; 5 Department of Dermatology, University of Regensburg, 93042 Regensburg, Germany

## Abstract

**Background:**

Epidermal nevi (EN) represent benign congenital skin lesions following the lines of Blaschko. They result from genetic mosaicism, and activating FGFR3 and PIK3CA mutations have been implicated.

**Case presentation:**

We report a female patient with a systemic keratinocytic nevus also involving the oral mucosa. Molecular genetic analysis revealed a mosaicism of the *FGFR3 *hotspot mutation R248C in the EN lesions of the skin and of the oral mucosa. The detection of the R248C mutation in a proportion of blood leukocytes and a slight scoliosis suggest an EN syndrome.

**Conclusions:**

Our results show that activating *FGFR3 *mutations can also affect the oral mucosa and that extracutaneous manifestations of EN syndrome can be subtle. We highlight the theoretical risk of the patient having an offspring with thanatophoric dysplasia as gonadal mosaicism for the R248C mutation cannot be excluded.

## Background

Epidermal nevi (EN) are benign hamartomas of the skin arising from the embryonic ectoderm. Depending on the involved components of the epidermis, EN are further divided into organoid and non-organoid (keratinocytic) types [1]. They are usually present at birth or develop during the first years of life, and their incidence is estimated to be 1-3 per 1000 live births [2]. Keratinocytic nevi typically follow the lines of Blaschko. Systemic keratinocytic nevi are characterized by an extensive involvement of large skin areas and may be associated with skeletal, cerebral or ocular abnormalities, resulting in various types of EN syndromes [1,2]. EN represent genetic mosaicism of the skin and activating *FGFR3 *(Fibroblast Growth Factor Receptor 3) and *PIK3CA *point mutations have recently been identified in keratinocytic nevi [3-6].

We report a patient with systemic EN associated with a slight scoliosis, who displayed mosaicism of the R248C *FGFR3 *mutation in epidermis, oral mucosa and blood leukocytes.

## Case presentation

**Figure 1:**
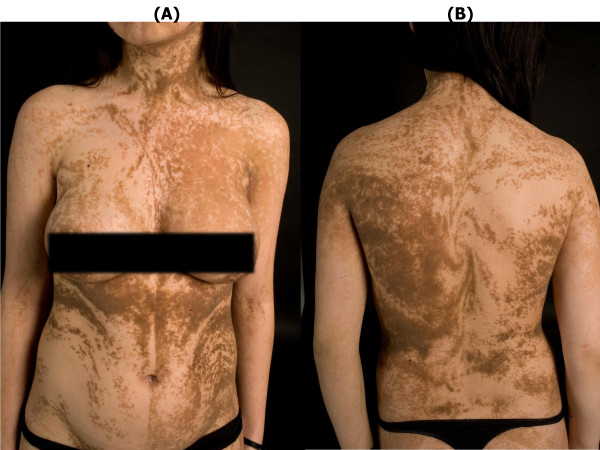
**17-year old woman with an extensive, systemic epidermal nevus following the lines of Blaschko**.

**Figure 2:**
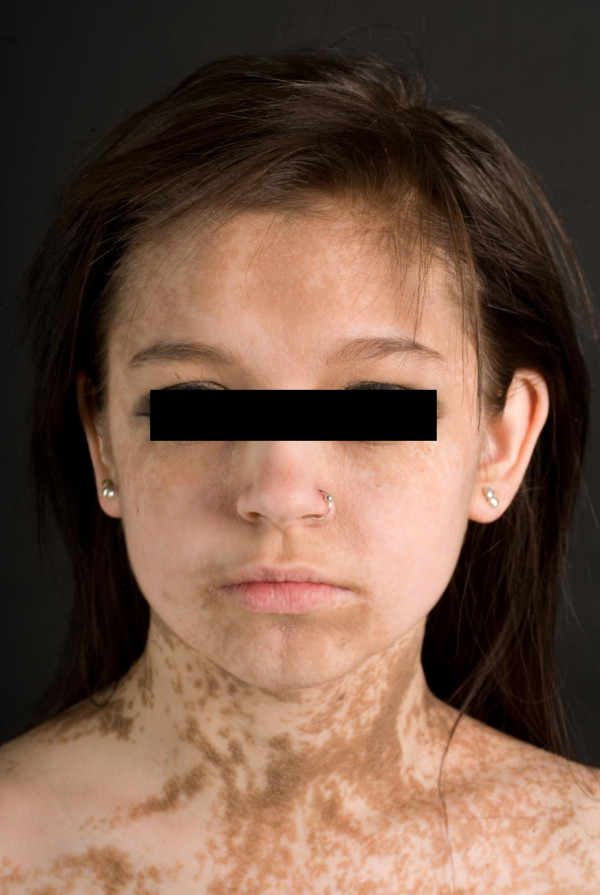
**Facial and neck involvement of epidermal nevus**.

**Figure 3:**
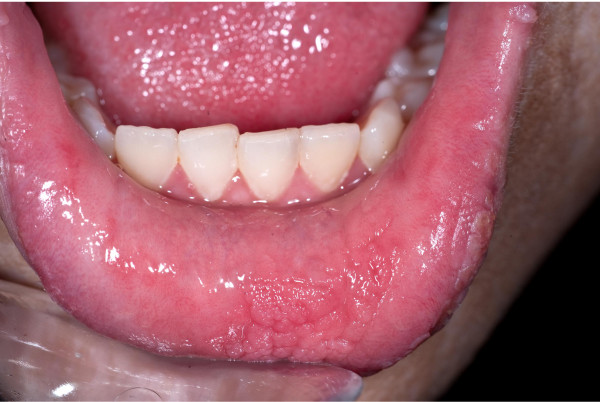
**Mucosal involvement of epidermal nevus**.

A 17-year old girl was referred with widespread EN (Figure 1 A+B). She was otherwise healthy apart from a tendency to back pain. Her parents recalled the first appearance of the EN when she was 4 months old. The EN initially presented as hyperpigmented linear streaks which gradually increased in size and thickness, becoming more elevated and verrucous. The brown, papillomatous and velvety EN followed the lines of Blaschko, with streaks and whorls on her body stopping abruptly at the ventral midline. The EN extended to her neck, scalp and extremities and was present on her face (Figure 2). She had intraoral mammilated lesions inside her lower lip (Figure 3) and at the buccal mucosa close to her oral angles. Laterally at the hard palate she had cobblestone-like thickening of the mucosa. She did not show any dysmorphic features and her face, trunk and extremities appeared symmetric with normal proportions, although a radiologic examination of the spine revealed a minimal thoracic scoliosis of 5 degrees. An eye examination was unremarkable and neurological examination was normal.

After informed consent of the patient and her parents, a 4 mm punch biopsy was taken from the chest. On histological examination, the biopsy showed a slightly papillomatous surface with non-specific laminated hyperkeratosis and acanthosis, typical of EN.

The patient was diagnosed with a bilateral, systemic keratinocytic nevus of the non-epidermolytic subtype. Maceration in the intertriginous areas was troublesome, but repeated laser (carbondioxide and Nd:YAG) treatments were without great success, as the skin lesions either relapsed or formed disfiguring scars.

## Genetic analysis

**Figure 4:**
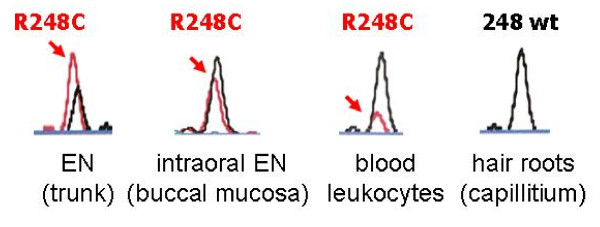
**Genetic analysis of the *FGFR3 *gene by a SNaPshot^® ^assay in various tissues revealed a mosaicism of the R248C hotspot mutation**.

Skin biopsies were taken from the EN on the abdomen and from adjacent normal skin after informed consent of the patient according to the guidelines of the local ethics committee and the Declaration of Helsinki. Separate fibroblast cultures were established from these biopsies. DNA was extracted directly from the skin biopsies as well as from cultured fibroblasts. In addition, formalin-fixed paraffin-embedded biopsy material, blood leukocytes, buccal brushings from lesional mucosa, scalp hair roots, and urothelial cells from urine sediment were available for analysis (Table 1). DNA was extracted from these tissues and cells using standard protocols. *FGFR3 *and *PIK3CA *mutations were analyzed using SNaPshot^® ^assays as described previously [6,7]. We identified the *FGFR3 *hotspot mutation R248C in EN tissue, but not in the adjacent normal skin (Figure 4). The R248C mutation was also detected in the EN tissue of the buccal mucosa harvested by buccal brushings. In contrast, the R248C mutation was not found in cultured fibroblasts from either affected or normal skin, nor in hair roots from affected skin of the scalp or in the urothelial cells. No mutations in the *PIK3CA *gene were found in any of the tissue samples.

**Table 1:** Results of genetic analysis

Sample	Localization	*FGFR3*	*PIK3CA*
1 EN	Trunk	R248C	wt
2 EN	Trunk	R248C	wt
3 Adjacent normal skin	Trunk	wt	wt
4 Cultured fibroblasts (EN tissue)	Trunk	wt	wt
5 Cultured fibroblasts (normal skin)	Trunk	wt	wt
6 Hair roots	Scalp	wt	na
7 Intraoral EN	Buccal mucosa	R248C	na
8 Urothelial cells	Urine sediment	wt	na
9 Leukocytes	Blood	R248C/wt	wt

EN, epidermal nevus; *FGFR3*, Fibroblast Growth Factor Receptor 3; *PIK3CA*, Phosphatidylinositol 3-kinase Catalytic 110 kD Alpha subunit; wt, wildtype; na, not available.

Immunohistochemical staining of the EN tissue with FGFR3 antibody (Santa Cruz Biotechnology, Santa Cruz, USA) revealed expression of FGFR3 protein in the complete epidermal layer, but the staining intensity was comparable with a normal skin control.

The results indicate that the systemic keratinocytic nevus syndrome in the present patient is caused by mosaicism of the R248C *FGFR3 *mutation. This mosaicism has extended to the oral mucosa, and caused intraoral lesions. The genetic mosaicism can be limited to the epidermis, but may also extend to other tissues, resulting in EN syndromes [1,2]. The proportion of EN patients with additional abnormalities of other organ systems is not known, and may vary depending on the different subtypes of EN. One comprehensive study identified an EN syndrome in 33% of 119 EN patients [8].

In keratinocytic nevi, activating point mutations in *FGFR3 *and *PIK3CA *genes have been identified in about 40% of patients [5,6]. Interestingly, the mutational spectrum is restricted to hotspots, including R248C for *FGFR3 *and E545G for *PIK3CA*. The same mutations have been identified in sporadic malignant tumors, and confer oncogenic properties to cells *in vitro *[9,10]. A non-mosaic constitutional R248C *FGFR3 *mutation causes thanatophoric dysplasia, a typically lethal skeletal dysplasia [11]. Obviously, surviving patients with the R248C *FGFR3 *mutation must be mosaics [12].

*FGFR3 *and *PIK3CA *mutations have also been identified in seborrheic keratoses which are benign epidermal skin tumors arising in adult patients with an increasing age-related incidence [5,13-15]. Although the identified *FGFR3 *and *PIK3CA *mutations are oncogenic [10,16,17] and can activate downstream signalling pathways [18,19], neither EN nor seborrheic keratoses bear a significant risk of malignant transformation.

In the patient of this case report, the R248C *FGFR3 *hotspot mutation was identified in the EN tissue of the skin and buccal mucosa (ectoderm) as well as a substantial proportion of blood leukocytes (mesoderm). With the involvement of two embryologic tissues, one might conclude that the mutation had happened at a very early stage, in a cell whose descendant population included skin and hematogenous precursor cells. The early occurrence of the mutation might be the reason for the widespread skin involvement.

To the best of our knowledge, we could show for the first time that the mosaic *FGFR3 *mutation can be associated with intraoral EN lesions, an otherwise uncommon observation [20]. The wildtype sequence of unaffected skin, hair roots and cultured fibroblasts in our patient confirms that the R248C mutation occurred in a mosaic form restricted to the epithelium and absent from the stromal component of skin. We propose that this case represents an EN syndrome, given the presence of the mutation in blood leukocytes indicating bone marrow involvement. Even her slight scoliosis may be part of the syndrome, although the finding could be coincidental and independent from the mosaicism. Hitherto the R248C *FGFR3 *mutation has been identified in two cases of an EN syndrome: - one with facial dysmorphism, and the other patient with cerebral involvement [21,22]. A preliminary designation of "FGFR3 EN syndrome" has been proposed, while acknowledging that a genetic heterogeneity may underlie the clinical phenotype of this syndrome [1,22]. For patients with extensive EN, a work-up for EN syndrome is indicated even without obvious extracutaneous features, as in our case.

Furthermore, it is important to be aware that these patients may have a predisposition towards malignancies of internal organs, especially low grade urothelial carcinomas [23-25]. As yet, there has been no report identifying *FGFR3 *mutations in a patient with EN associated with urothelial cancer or other malignancies [26]. However, *FGFR3 *mutations are involved in the pathogenesis of bladder tumors, cervix carcinoma, and multiple myeloma [9,27]. The risk for the present patient is difficult to assess, although the absence of the *FGFR3 *mutation in urothelial cells suggests that at least for bladder cancer, it may be low. The risk for malignant transformation of an EN lesion (e.g., basal cell carcinoma or squamous cell carcinoma) appears likewise to be low, although occasional incidences have been reported [28-30].

If the *FGFR3 *mosaicism involves the gonad, our patient is at risk of having offspring with thanatophoric dysplasia. There is precedent for this, in a female patient with extensive mosaicism of the R248C *FGFR3 *mutation, including widespread acanthosis nigricans of the skin and skeletal dysplasia [31].

## Conclusions

We show that a mosaicism of the R248C *FGFR3 *mutation can cause a systemic EN with involvement of the oral mucosa. For patients with extensive EN, a work-up for "EN syndrome" is indicated, as extracutaneous manifestations of the EN syndrome can be subtle. As gonadal mosaicism for the R248C *FGFR3 *mutation cannot be entirely excluded, a risk of having offspring with thanatophoric dysplasia exists. Prenatal diagnosis for the R248C mutation will therefore be offered in future pregnancies of our patient.

## Consent

Written informed consent was obtained from the patient for publication of this case report and any accompanying images. A copy of the written consent is available for review by the Editor-in-Chief of this journal.

## Competing interests

The authors declare that they have no competing interests.

## Authors' contributions

AB was responsible for the clinical work-up and wrote the draft of the manuscript. CF performed the genetic counselling, arranged fibroblast cultures and DNA extraction from skin and blood, and participated in discussing the results. OC took part in the histopathological classification and discussion, and supplied material for DNA extraction from paraffin blocks and urine. BF participated in the genetic analysis. CH performed the genetic analyses, immunohistochemistry and participated in discussing the results and writing the manuscript. All authors read and approved the final manuscript.

## Acknowledgements and Funding

We thank Eva Herschberger for excellent technical support. This work was supported partly by a grant to C.H. from the Deutsche Forschungsgemeinschaft (DFG HA 5531/1).

## Pre-publication history

The pre-publication history for this paper can be accessed here:

http://www.biomedcentral.com/1471-2350/12/79/prepub
